# Incidence and Characteristics of Cerebellar Atrophy/Volume Loss in Children with Confirmed Diagnosis of Tuberous Sclerosis Complex

**DOI:** 10.3390/children11060627

**Published:** 2024-05-24

**Authors:** Livja Mertiri, Eugen Boltshauser, Stephen F. Kralik, Nilesh K. Desai, Maarten H. Lequin, Thierry A. G. M. Huisman

**Affiliations:** 1 Edward B. Singleton Department of Radiology, Texas Children’s Hospital and Baylor College of Medicine, Houston, TX 77030, USA; sfkralik@texaschildrens.org (S.F.K.); nkdesai@texaschildrens.org (N.K.D.); mxlequin@texaschildrens.org (M.H.L.); huisman@texaschildrens.org (T.A.G.M.H.); 2 Division of Pediatric Neurology, University Children’s Hospital Zurich, 8032 Zurich, Switzerland; eugen.boltshauser@bluewin.ch

**Keywords:** tuberous sclerosis complex, cerebellar atrophy, hippocampal sclerosis, subependymal giant cell astrocytomas, pediatric population

## Abstract

**Objectives:** The goal of our study was to determine the incidence of cerebellar atrophy, assess the imaging findings in the posterior fossa and determine the incidence of hippocampal sclerosis in a cohort of pediatric patients with confirmed tuberous sclerosis complex (TSC). **Material and methods:** MRI studies of 98 TSC pediatric patients (mean age 7.67 years) were evaluated for cerebellar atrophy, cerebral/cerebellar tubers, white matter lesions, subependymal nodules, subependymal giant cell astrocytomas, ventriculomegaly, and hippocampal sclerosis. Clinical charts were revisited for clinical symptoms suggesting cerebellar involvement, for seizures and treatment for seizures, behavioral disorders and autism. **Results:** Cerebral tubers were present in 97/98 cases. In total, 97/98 had subependymal nodules, 15/98 had SEGA, 8/98 had ventriculomegaly and 4/98 had hippocampal sclerosis. Cerebellar tubers were found in 8/98 patients (8.2%), whereas cerebellar atrophy was described in 38/98 cases (38.8%). In 37/38 patients, cerebellar volume loss was mild and diffuse, and only one case presented with left hemi-atrophy. Briefly, 32/38 presented with seizures and were treated with anti-seizure drugs. In total, 8/38 (21%) presented with behavioral disorders, 10/38 had autism and 2/38 presented with seizures and behavioral disorders and autism. **Conclusions:** Several studies have demonstrated cerebellar involvement in patients with TSC. Cerebellar tubers differ in shape compared with cerebral tubers and are associated with cerebellar volume loss. Cerebellar atrophy may be focal and diffuse and one of the primary cerebellar manifestations of TSC, especially if a TSC2 mutation is present. Cerebellar degeneration may, however, also be secondary/acquired due to cellular damage resulting from seizure activity, the effects of anti-seizure drugs and anoxic–ischemic injury from severe seizure activity/status epilepticus. Further, prospective studies are required to identify and establish the pathogenic mechanism of cerebellar atrophy in patients with TSC.

## 1. Introduction

Tuberous sclerosis complex (TSC) is an autosomal-dominant neurocutaneous syndrome characterized by the formation of hamartomas in multiple organs, including the brain, skin, retina, heart, kidneys, and lungs [1]. It results from inactivating mutations of either the TSC1 or TSC2 genes on chromosome 9p and 16q encoding for the proteins hamartin and tuberin, respectively [2,3]. Mutations of these tumor suppressor genes lead to excessive translation of proteins associated with cell growth [4]. About 30% of TSC cases have been attributed to TSC1 mutation, 70% have been attributed to TSC2 mutation, and in around 5–10% of cases, a known mutation has not been found [5].

CNS involvement is the most common cause of morbidity and mortality in TSC patients. Clinically, they may present with seizures, intellectual disability, autism spectrum disorders and other behavioral and neurodevelopmental impairments [6]. Major brain structural abnormalities consist of cortical and subcortical tubers, white matter lesions, subependymal nodules (SENs) and subependymal giant cell astrocytomas (SEGAs) [6].

Tubers are characteristic findings and are found in greater than 80% of patients with TSC [6]. Cerebral tubers vary in size, number and location, and most of them are located in the frontal and parietal lobes [7]. They can be either cortical or subcortical, and are typically centered on the cortex with their apex oriented towards the ventricles following the shape of the gyri, suggesting abnormal neuronal migration [8].

Cerebellar tubers are less common, with a reported prevalence between 9% and 30% [8,9], and most of them are associated with TSC2 gene mutations [8]. Usually, they are not found in absence of cerebral tubers [9], representing a powerful predictor of supratentorial cortical involvement. Cerebellar tubers occur with older age, and usually are localized in the posterior cerebellar lobes that control the cognitive, autonomic and affective functions. Their location may explain the association with neurobehavioral disorders and the absence of cerebellar motor signs in these patients [10].

Furthermore, several studies have demonstrated TSC to be associated with cerebellar volume loss that is not otherwise observed in supratentorial tubers [11]. It may be focal or diffuse, with the resultant widening of the cerebellar fissures.

Abnormalities of the hippocampus are not usually grouped with the typical structural lesions of TSC. To our knowledge, few studies have described the coexistence of hippocampal sclerosis in patients with TSC. Gama et al., in a series of cases with TSC, demonstrated the presence of hippocampal abnormalities, also confirming that those patients had a tendency to exhibit more cortical tubers [12].

The goal of our study was to determine the incidence of cerebellar atrophy, assess the imaging findings in the posterior fossa and determine the incidence of hippocampal sclerosis in a cohort of pediatric patients with confirmed TSC.

## 2. Materials and Methods

We retrospectively reviewed 98 MRI scans performed in our tertiary pediatric hospital between July 2022 and July 2023. The age of these patients ranged from 1 day to 24 years (mean age: 7.67 years). Study data were collected using a computer-assisted search of all radiological reports using the keywords “tuberous sclerosis complex”. In all MRI scans, a standard departmental protocol was applied using multiplanar pre- and post-contrast T1-weighted sequences as well as T2-weighted sequences, fluid attenuated inversion recovery and diffusion tensor imaging. All patients met the clinical and radiological criteria necessary for a definitive diagnosis of TSC [13]. Patients without obvious TSC pathology on MR imaging or with unavailable clinical records were excluded from the analysis.

Collected parameters included demographic characteristics, clinical and MR imaging findings and genetic testing results for TSC1/2 gene mutations.

All scans were systematically evaluated for the presence, number and location of supratentorial and infratentorial lesions by two readers (LM and TH). In particular, the assessed MRI findings included the presence of cerebral tubers (cortical and subcortical), white matter lesions, SENs, SEGAs, and hippocampal sclerosis. On MR imaging, tubers were defined as T1-hypointense, and T2- and FLAIR-hyperintense focal lesions with a reversed MRI pattern in young infants due to ongoing myelination [6]. Tubers may expand the gyri/folia and blur the margin between the gray and white matter [14]. White matter lesions were defined as T1-hypointense, and T2- and FLAIR-hyperintense lesions that may demonstrate three distinctive patterns: curvilinear bands extending from the periventricular white matter towards the cortex, wedge-shaped lesions, and cerebellar radial bands [14]. SENs were defined as small, irregular hamartomatous nodules along the ependymal surface of the ventricles. They appear isointense-to-gray matter, but in 90% of cases, may calcify resulting in being T1- and T2-hypointense [6] on MRI. SEGAs are low-grade tumors typically localized at the level of the interventricular foramen of Monro causing obstructive hydrocephalus because of the mass effect around the ventricular system. They appear as focal T1-hypointense and T2-hyperintense lesions with strong enhancement on post-contrast images [6]. On MRI T2-weighted and FLAIR scans, hippocampal sclerosis was defined as a smaller-sized (atrophic) hippocampus with an increased signal and with a less distinct internal gray–white matter differentiation/structure [10].

In addition, images were assessed for cerebellar involvement, including cerebellar tubers and cerebellar atrophy, characterized by a widening of the cerebellar fissures and/or fourth ventricle [9].

In those patients with posterior fossa involvement on imaging, the clinical charts were evaluated for symptoms suggesting cerebellar involvement, seizures, anti-seizure treatment, and neurobehavioral disorders.

## 3. Results

In 16/98 cases (16%), a TSC1 gene mutation was identified, while 57/98 patients (58%) had a TSC2 mutation. In six cases, TSC1 and TSC2 mutations were not detected, and in two cases, genetic testing results were pending. For the remaining 17 cases, genetic testing was either not performed or data were unavailable.

Cerebral tubers were present in 97/98 cases (99%), with SEN observed in 97/98 cases. In total, 15/98 (15%) presented with SEGAs, while ventriculomegaly was noted in 8/98 (8%) cases, and hippocampal sclerosis was seen in 4/98 (4%) cases.

Cerebellar tubers were found in 8/98 patients (8.2%), with 6 of them being single and 2 being multifocal. Among these, 6 were localized in the right hemisphere and 2 were in the left hemisphere. Four of eight cases with cerebellar tubers (two single and two multifocal) were associated with mild diffuse cerebellar atrophy. 

Cerebellar atrophy was described in 38/98 cases (38.8%), with 37 of them exhibiting mild and diffuse atrophy, and only 1 case showing left hemi-atrophy. Among those with cerebellar atrophy, 32 presented with seizures and were treated with anti-seizure drugs. Additionally, 8/38 (21%) presented with behavioral disorders, while 10/38 had autism. In total, 2/38 presented with seizures, behavioral disorders and autism.

## 4. Discussion

**Figure 1:**
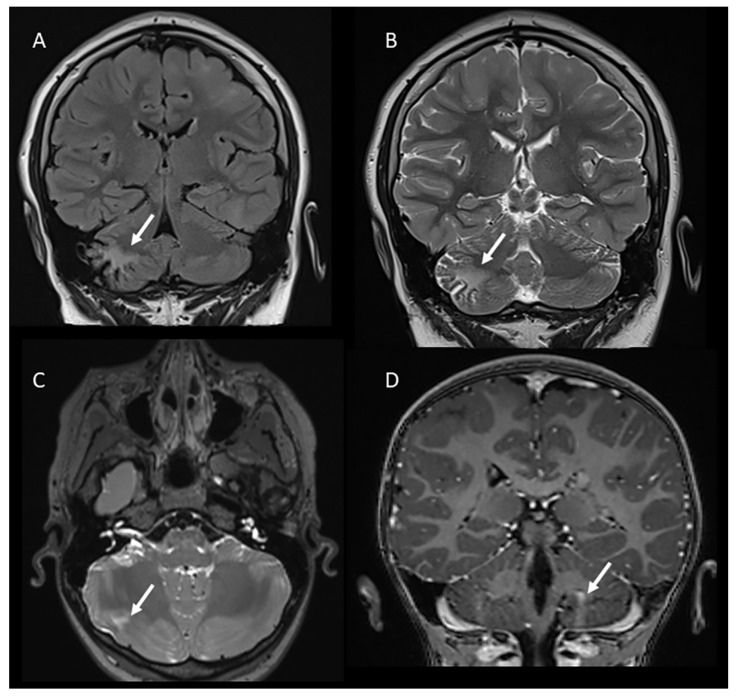
Coronal FLAIR (**A**) and T2-weighted (**B**) images demonstrating a cerebellar tuber in the right hemisphere in a 13-year-old male. Axial T2-weighted (**C**) and coronal post-contrast T1-weighted images (**D**) showing other examples of cerebellar tubers (arrows) in two patients with tuberous sclerosis (8 and 5 years old, respectively).

Supratentorial tubers are seen in 80–95% of patients with TSC, while cerebellar involvement is less frequent and occurs in 10–44% of patients according to previous data [8,9,15]. However, in studies focused on pediatric populations, the incidence is slightly lower, and cerebellar tubers are primarily seen in patients over 11.5 years of age and are rare in patients under the age of 8 years [11]. In our cohort (mean age 7.67 years), the incidence of cerebellar tubers was 8/98 (8.2%) patients, matching the results of the previously reported studies. The majority of the encountered cerebellar tubers were single (6/8) and localized in the right hemisphere (6/8) (Figure 1).

MR imaging characteristics of cerebellar tubers have been reported to be similar to those of supertentorial tubers, although with a difference in shape due to the specific neuronal migration within the cerebellum, which differs from the supratentorial brain. In particular, Ertan et al. noted that cerebellar tubers are typically wedge-shaped with a broad base oriented towards the cerebellar cortex due to the migration of cell neurons from the external surface inward past the Purkinje cells [9].

Although no mechanism of causation has been established, patients with cerebellar tubers are known to show high association with TSC2 mutations [16], and this was also confirmed by our results (six out of eight patients presented with a TSC2 gene mutation, while in two out of eight patients, no TSC gene mutations were detected).

Cerebellar atrophy has previously been reported in case reports and small series of patients with TSC. Previous studies have described focal volume loss, typically associated with cerebellar tubers due to the gliotic process within the tubers, which may lead to both atrophy and calcifications [17].

Other studies report more diffuse volume loss, with the involvement of the entire cerebellum, particularly for those with TSC2 mutations [4]. TSC2 plays a critical role in Purkinje cell survival by regulating endoplasmatic reticulum and oxidative stress; therefore, loss-of-function mutations of this gene lead to cell death from apoptosis and result in diffuse cerebellar volume loss [2,18].

**Figure 2:**
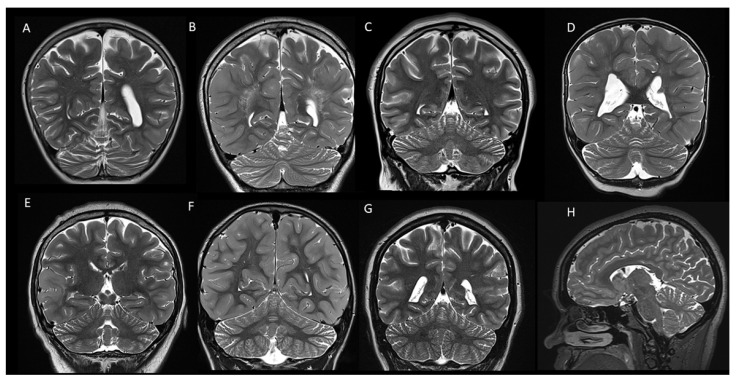
Coronal (**A**–**G**) and sagittal (**H**) T2-weighted images of 8 patients with tuberous sclerosis demonstrating mild diffuse cerebellar atrophy with widening of the cerebellar fissures.

**Figure 3:**
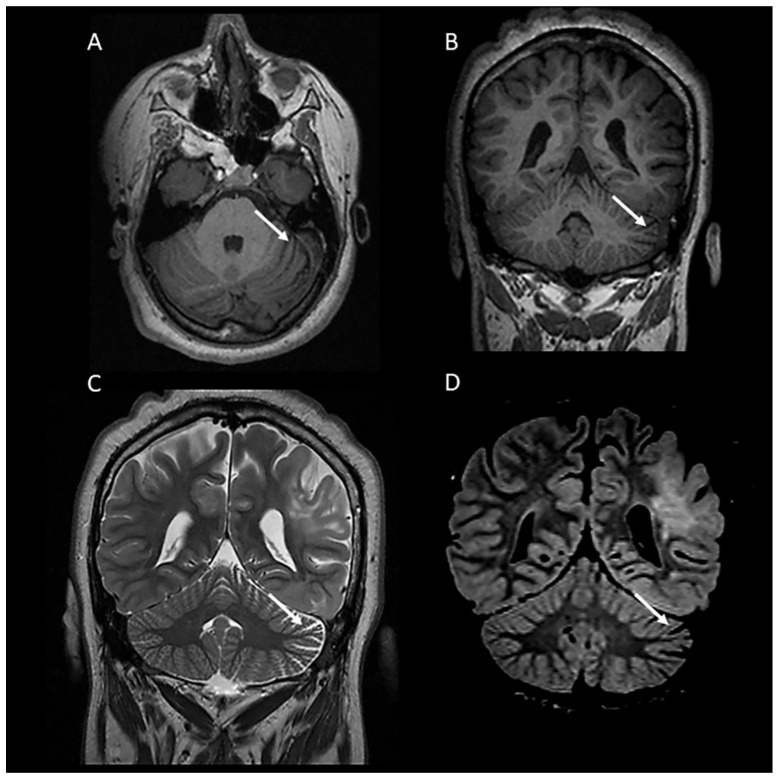
A 19-year-old female with tuberous sclerosis complex. Axial (**A**) and coronal (**B**) T1-weighted and coronal T2-weighted (**C**) and FLAIR (**D**) MR images demonstrating left hemi-cerebellar atrophy characterized by unilateral widening of the cerebellar fissures.

In our cohort, 38/98 cases (38.8%) presented with mild diffuse cerebellar atrophy, characterized by a widening of the cerebellar fissures and/or the fourth ventricle (Figure 2). Interestingly, one case presented with left hemi-cerebellar atrophy without any involvement of the other cerebellar hemisphere (Figure 3). Four out of eight cases with cerebellar tubers (two single and two multifocal) were associated with mild diffuse cerebellar atrophy. Three of them presented with TSC2 gene mutation, confirming the high association of this mutation with cerebellar tubers and cerebellar volume loss, and in one case, no mutations were detected on TSC1/2 genetic testing.

Cerebellar degeneration can also be attributed to cellular damage from seizure activity, the effects of anti-seizure drugs and anoxic–ischemic injury from prolonged severe seizures [19].

Several studies have demonstrated the correlation between cerebellar atrophy and phenytoin toxicity in patients with epilepsy [20,21]. Phenytoin was a widely used anti-seizure drug for controlling both generalized and partial seizures. The chronic effects of phenytoin are often difficult to discriminate from those of the seizures, but have been recognized, whether in the therapeutic range or not, as an important factor of cerebellar degeneration [20,21].

In our study, among the 38 patients with TSC-associated cerebellar atrophy, 32 had seizures and used anti-seizure drugs, but none of them were treated with phenytoin. The most used anti-seizure drugs included Diazepam, Clonazepam, Oxcarbazepine, Vigabatrin and Levetiracetam.

Remarkably, TSC patients with cerebellar involvement may not present motor symptoms [9,15,18]. Although traditionally the cerebellum is considered a major contributor to motor function and in particular coordination, it is also involved in cognitive, affective, and language function. In our study, 26/98 (26%) cases among the entire study group and 8/38 (21%) among those with cerebellar atrophy presented with behavioral impairments. In total, 22/98 (22%) and 10/38 (26.3%) had autism spectrum disorders, respectively. This is consistent with several published studies in which children with TSC-associated cerebellar lesions presented with cognitive and neurobehavioural disorders, along with motor disabilities. Toldo et al. demonstrated that TSC-associated cerebellar lesions are more frequently localized in the posterior cerebellar lobe and have a significant correlation with a more severe clinical and neuroradiological phenotype due to association with TSC2 mutations, but this is not considered to be the best predictor of neurobehavioral outcomes [10]. Eluvathingal et al. also reported an overall higher incidence of autistic symptomatology in TSC patients, in particular in those with right-sided cerebellar involvement [15]. However, further studies are mandatory to determine the correlation between cerebellar involvement in TSC patients and the incidence of developmental delay.

Epilepsy is the most prevalent clinical manifestation in TSC, occurring in 80–90% of patients [22]. Hippocampal abnormalities are also a well-known cause of seizures and mainly manifest as mesial temporal sclerosis with partial complex seizures [12]. Few studies have reported hippocampal abnormalities in patients with TSC. Lang et al. reported a case of a 7-month-old boy with intractable seizures who was diagnosed with TSC2 mutation. Imaging showed diffuse cerebral volume loss with multiple cerebral tubers and hippocampal CA1 sclerosis [23]. In another published study, hippocampal abnormalities were described in five patients with TSC [12]. In our cohort, four patients showed hippocampal abnormalities on MRI and clinically, and all of them presented with seizures.

**Figure 4:**
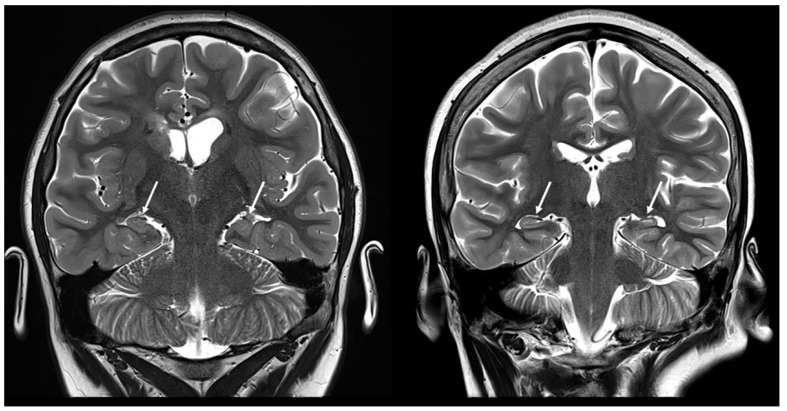
Coronal T2-weighted images demonstrating hippocampal abnormalities in two patients with tuberous sclerosis (10 and 12 years old, respectively).

Hippocampal sclerosis could be considered an incidental finding; however, it may also be a secondary complication/result of a multifactorial process (Figure 4). Two different mechanisms have been hypothesized to explain its pathology. First, hippocampal injury may result from recurrent epileptic activity in TSC that damages the anatomic connections within the temporal lobe [24]. The second hypothesis is that there is a common pathological differentiation between the proliferation of germinal matrix cells and the migrational arrest of altered neurons that lead to both cortical tubers and hippocampal sclerosis in TSC [25].

Several limitations are present in this study. This study had a retrospective design; no follow up examinations and no prospective examination of the symptomatology were performed. Although this study is one of the largest series analyzed to date, these preliminary data show that future prospective studies are mandatory. In addition, secondary to the retrospective nature of this preliminary study, no reliable correlation with the frequency and duration of seizures could be established. Similarly, no systematic correlation between cerebellar atrophy and medication could be studied. Additionally, the failure to analyze the relationship between patients’ age and cerebellar lesions (tuber and atrophy) represents a limitation of this study, and highlights an important area for future investigation. Future studies should address these open questions.

## 5. Conclusions

Several studies have demonstrated cerebellar involvement in patients with TSC. Cerebellar tubers differ in shape compared with supratentorial tubers and are associated with cerebellar volume loss that is not otherwise observed in supratentorial tubers. In our large cohort of pediatric patients, cerebellar atrophy was seen in 38.8% of children diagnosed with TSC. Cerebellar atrophy may be focal and diffuse, and the frequently described association with TSC2 mutations may explain the pathogenesis, suggesting that cerebellar atrophy could be one of the manifestations of the TSC syndrome. Additionally, cerebellar degeneration may result from cellular damage due to seizure activity, the effects of anti-seizure drugs and anoxic—ischemic injury from seizures. Further, prospective studies are required to identify and establish the pathogenic mechanism of cerebellar atrophy in patients with TSC. Furthermore, longitudinal studies may be indicated to confirm that the small size of the cerebellum was indeed a result of atrophy and not secondary to cerebellar hypoplasia or, possibly, a combination of hypoplasia and atrophy.

## Data Availability

The original contributions presented in the study are included in the article, further inquiries can be directed to the corresponding author.

## Abbreviations

FLAIR: Fluid Attenuated Inversion Recovery, MRI: Magnetic Resonance Imaging, SEN: Subependymal Nodules, TSC: Tuberous Sclerosis Complex, SEGA: Subependymal Giant Cell Astrocytoma.
